# Primary adrenal small cell neuroendocrine carcinoma in a patient with sarcoidosis and synchronous colon adenocarcinoma

**DOI:** 10.1210/jcemcr/luag313

**Published:** 2026-10-13

**Authors:** Asami Sano, Tetsu Takeichi, Akio Miyamaoto, Yutaka Nishii, Teppei Yamamoto, Yukiko Kusama

**Affiliations:** Department of Diabetes, Endocrinology and Metabolism, Nagano Municipal Hospital, Nagano 381-0006, Japan; Department of Diabetes, Endocrinology and Metabolism, Nagano Municipal Hospital, Nagano 381-0006, Japan; Department of Diabetes, Endocrinology and Metabolism, Nagano Municipal Hospital, Nagano 381-0006, Japan; Department of Diabetes, Endocrinology and Metabolism, Nagano Municipal Hospital, Nagano 381-0006, Japan; Department of Urology, Nagano Municipal Hospital, Nagano 381-0006, Japan; Department of Diagnostic Pathology, Nagano Municipal Hospital, Nagano 381-0006, Japan

**Keywords:** adrenal incidentaloma, adrenalectomy, chromogranin A, cisplatin, ascending colon, positron emission tomography/computed tomography

## Abstract

Primary adrenal neuroendocrine carcinoma (NEC), particularly the small cell subtype, is exceptionally rare. In patients with sarcoidosis, inflammatory lymphadenopathy may mimic a metastatic disease, making disease detection difficult. A 76-year-old man with sarcoidosis was referred for an enlarging left adrenal incidentaloma that had increased in size from 7 mm to 19 × 14 mm over 3 years. Hormonal evaluation revealed unremarkable findings; laparoscopic adrenalectomy was performed because malignancy could not be excluded. Histopathological examination revealed small cell NEC with positive synaptophysin and chromogranin A staining, and a Ki-67 labeling index of 80%. One month after surgery, para-aortic lymphadenopathy was detected. Fluorodeoxyglucose positron emission tomography/computed tomography (CT) showed uptake in the para-aortic lymph nodes and ascending colon, where colonoscopy identified synchronous adenocarcinoma. The cause of the lymphadenopathy was uncertain because of coexisting sarcoidosis and colon cancer. Cisplatin plus irinotecan induced a partial response; however, disease progression was observed. CT-guided lymph node biopsy confirmed metastatic NEC. Second-line amrubicin therapy reduced tumor size; comprehensive genomic profiling revealed a tumor mutation-burden-high phenotype. This case highlights diagnostic challenges in the evaluation of lymphadenopathy in patients with sarcoidosis and synchronous malignancy, emphasizing the importance of pathological confirmation for guiding appropriate management.

## Introduction

Primary adrenal neuroendocrine carcinoma (NEC) is an extremely rare malignancy; reports of its small cell subtype are particularly limited [1]. High-grade NECs are characterized by a high proliferative capacity, rapid progression, and poor prognosis [2]. Although adrenal lesions are frequently incidentally detected during imaging studies, distinguishing benign from malignant lesions and differentiating primary from metastatic tumors based solely on imaging findings is often challenging [3, 4]. In addition, sarcoidosis may manifest with fluorodeoxyglucose (FDG)-avid lymphadenopathy on positron emission tomography (PET)/computed tomography (CT), which can mimic lymph node metastases from malignant tumors [5, 6]. The coexistence of multiple malignancies may further complicate the interpretation of the primary tumor origin and metastatic patterns.

We report a case of primary adrenal small cell NEC that was initially detected as an enlarging adrenal incidentaloma and diagnosed following adrenalectomy. Interpreting postoperative para-aortic lymphadenopathy was challenging because of concomitant sarcoidosis-associated abdominal lymph node involvement and synchronous ascending colon adenocarcinoma. During systemic chemotherapy, CT-guided lymph node biopsy confirmed metastatic adrenal NEC. Herein, we describe the diagnostic and therapeutic challenges encountered in this rare case and present a review of the relevant literature.

## Case presentation

A 76-year-old man was referred to our department for evaluating a left adrenal mass. He had been undergoing regular radiological follow-up every 12 months for sarcoidosis at the Department of Respiratory Medicine. A 7-mm left adrenal nodule was identified in 2021, with an unenhanced attenuation of 43.9 Hounsfield units (HU). The lesion gradually enlarged over time, reaching 19 × 14 mm in February 2024, with an unenhanced attenuation of 43.8 HU. The patient was asymptomatic, and physical examination revealed no abnormalities. His medical history was remarkable for the presence of sarcoidosis. No family history of endocrine or malignant diseases was noted.

## Diagnostic assessment

Endocrinological evaluation revealed no evidence of hormone excess (Table 1). The serum adrenocorticotropic hormone, aldosterone, and catecholamine levels were within normal limits. Although the serum cortisol level measured at 14:00 was mildly below the reference range (4.9 μg/dL [SI: 135 nmol/L]) (reference range, 7.1-19.6 μg/dL [SI: 196-541 nmol/L]), the patient exhibited no clinical symptoms or signs suggestive of adrenal insufficiency, such as hypotension or electrolyte imbalances. The serum pro-gastrin-releasing peptide (proGRP) and soluble interleukin-2 receptor (sIL-2R) levels were mildly elevated, possibly reflecting subclinical neuroendocrine tumor activity and a history of sarcoidosis, respectively. Based on these findings, the lesion was initially diagnosed as a suspected nonfunctioning adrenal tumor. A repeat abdominal CT performed in July 2024 demonstrated further enlargement of the lesion to 24 × 23 mm (Fig. 1). The lesion showed an unenhanced attenuation of 36.7 HU and heterogeneous enhancement in the early contrast-enhanced phase (82.2 HU), followed by a decrease in attenuation to 70.8 HU in the delayed phase. Metaiodobenzylguanidine scintigraphy revealed no abnormal uptake in the adrenal mass.

**Figure 1:**
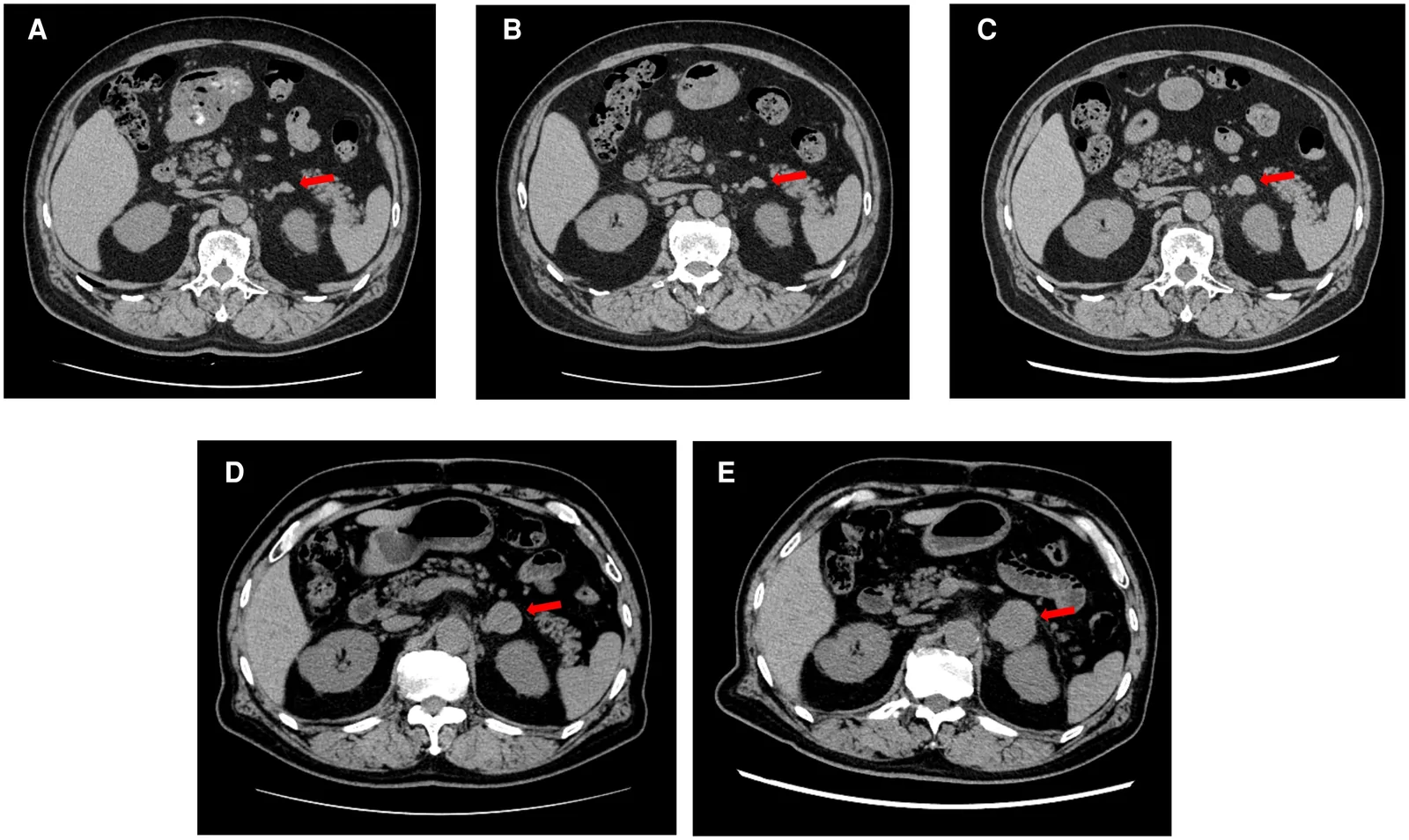
Serial computed tomography images demonstrating progressive enlargement of the left adrenal tumor. (A) February 2022. (B) February 2023. (C) February 2024. (D) July 2024. (E) December 2024. Arrows indicate the left adrenal tumor.

**Table 1:** Laboratory findings at the time of initial evaluation

Test	Value	Reference range
Cortisol (14:00)	4.9 μg/dL (SI: 135 nmol/L)	7.1-19.6 μg/dL (SI: 196-541 nmol/L)
ACTH	31.5 pg/mL (SI: 6.9 pmol/L)	7.2-63.3 pg/mL (SI: 1.6-13.9 pmol/L)
Aldosterone (immunoassay)	34.7 pg/mL (SI: 96 pmol/L)	4-82.1 pg/mL (SI: 11-228 pmol/L)
Plasma renin activity	1.2 ng/mL/h (1.2 ng/mL/hr)	0.2-2.3 ng/mL/h (SI: 0.2-2.3 ng/mL/h)
DHEA-S	774 ng/mL (SI: 2.10μmol/L)	50-2530 ng/mL (SI: 0.14-6.87 μmol/L)
Urinary metanephrine	0.24 μg/mg Cre	NA
Urinary normetanephrine	0.32 μg/mg Cre	NA
ACE	14.4 IU/L (14.4 IU/L)	7-25 IU/L (SI: 7-25 IU/L)
sIL2-R	591 U/mL (591 U/mL)	145-519 U/mL (SI: 145-519 U/mL)
proGRP (before chemotherapy)	99.3 pg/mL (99.3 pg/mL)	0-74.7 pg/mL (SI: 0-74.7 pg/mL)

Abbreviations: ACE, angiotensin-converting enzyme; ACTH, adrenocorticotropic hormone; DHEA-S, dehydroepiandrosterone sulfate; proGRP, pro-gastrin-releasing peptide; sIL-2R, soluble interleukin-2 receptor.

## Treatment

Progressive enlargement of the adrenal lesion raised concerns about malignancy, therefore, laparoscopic left adrenalectomy was performed in January 2025. Although the preoperative afternoon cortisol level was mildly low, the contralateral right adrenal gland was radiologically normal; the patient underwent the procedure uneventfully, without any perioperative adrenal insufficiency. Histopathological examination revealed diffuse nests of small atypical cells with hyperchromatic nuclei and scant cytoplasm. Immunohistochemical staining showed positivity for synaptophysin and chromogranin A. The Ki-67 labeling index was approximately 80%, consistent with a highly proliferative neoplasm. Based on these findings, the tumor was diagnosed as small cell NEC (Fig. 2).

**Figure 2:**
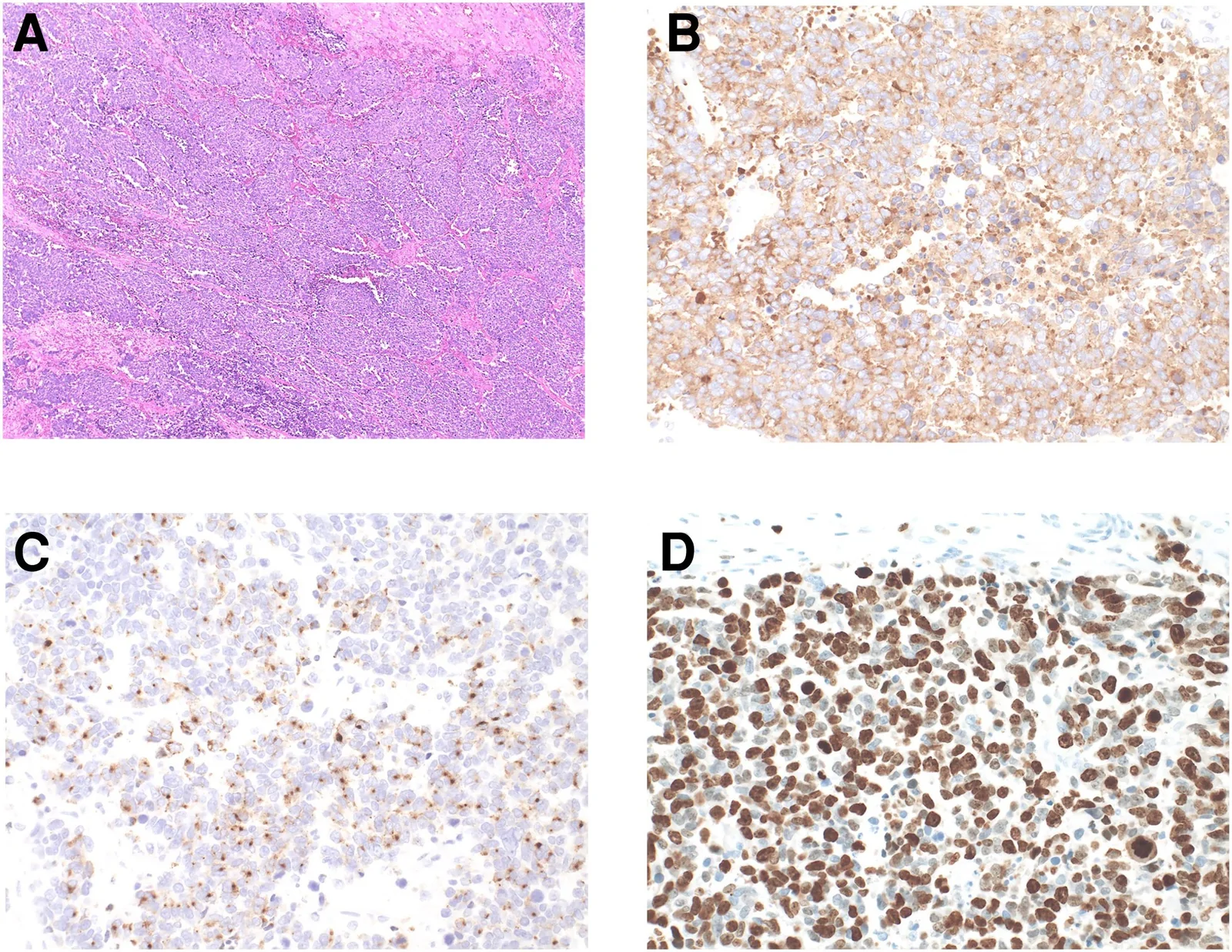
Histopathological findings of the adrenal tumor. (A) Hematoxylin and eosin staining showing diffuse proliferation of atypical tumor cells (×40). (B) Positive staining for synaptophysin (×200). (C) Positive staining for chromogranin A (×200). (D) Ki-67 labeling index of approximately 80% (×200).

One month after surgery, contrast-enhanced CT revealed enlargement of the para-aortic lymph nodes. FDG-PET/CT revealed intense FDG uptake in the para-aortic lymph nodes and ascending colon (Fig. 3). Colonoscopy demonstrated a neoplastic lesion in the ascending colon, and biopsy confirmed adenocarcinoma. Chest CT showed no pulmonary lesions suggestive of primary small cell lung cancer or other thoracic malignancies.

**Figure 3:**
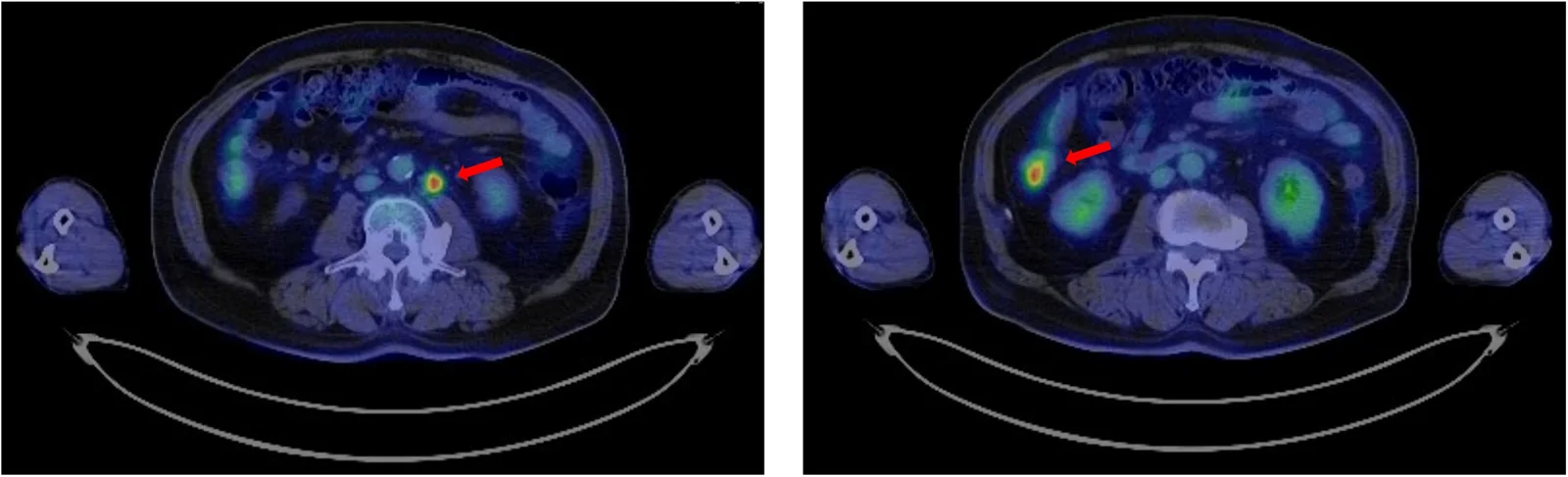
Fluorodeoxyglucose positron emission tomography/computed tomography findings after adrenalectomy. Arrows indicate FDG accumulation in the lymph node and ascending colon.

Interpreting the para-aortic lymphadenopathy was challenging because the patient had preexisting sarcoidosis-associated abdominal lymphadenopathy and synchronous ascending colon adenocarcinoma. However, the serum angiotensin-converting enzyme (ACE) and sIL-2R levels remained stable, and the distribution of lymphadenopathy was atypical for metastatic spread from the ascending colon cancer. The highly aggressive pathological features of adrenal NEC supported the clinical diagnosis of metastatic adrenal NEC. Consequently, systemic chemotherapy with cisplatin and irinotecan was initiated in April 2025.

## Outcome and follow-up

After 2 cycles of cisplatin plus irinotecan, follow-up CT demonstrated a reduction in the size of the para-aortic lymph nodes, accompanied by a decrease in the serum proGRP levels. However, the proGRP levels subsequently increased, and CT performed after 6 cycles revealed enlargement of the para-aortic lymph nodes, indicating disease progression (Fig. 4).

**Figure 4:**
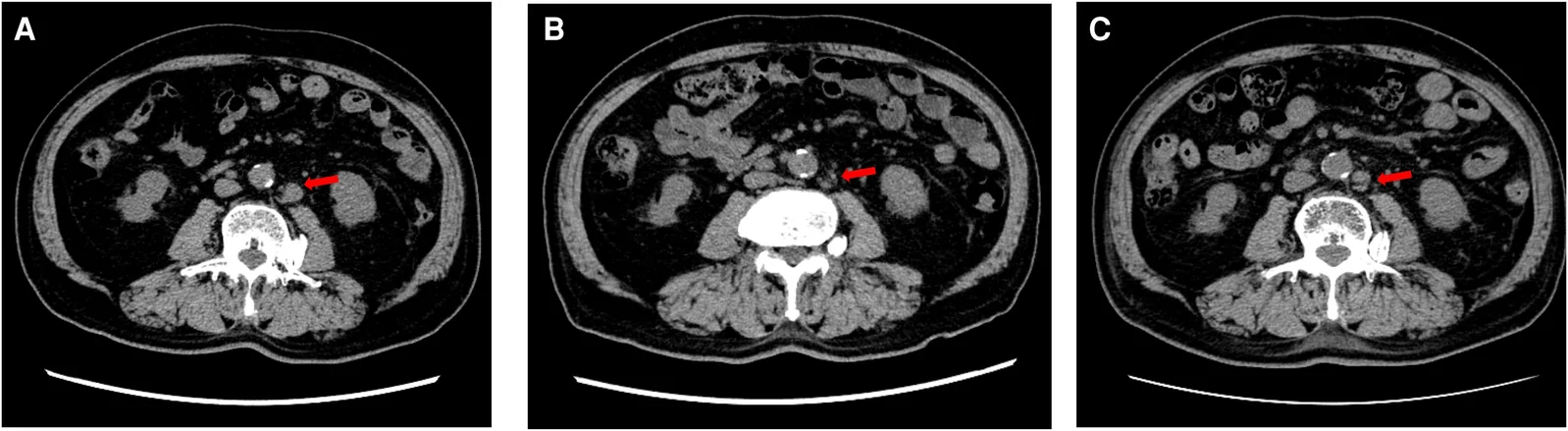
Radiological course of para-aortic lymph node metastases during chemotherapy. (A) Before chemotherapy; (B) after 2 cycles of cisplatin plus irinotecan; and (C) after 6 cycles of cisplatin plus irinotecan. Arrows indicate the enlarged para-aortic lymph nodes.

To distinguish recurrent NEC from sarcoidosis exacerbation, CT-guided biopsy of a para-aortic lymph node was performed. Histopathological examination confirmed the presence of metastatic NEC. Therefore, second-line chemotherapy with amrubicin was initiated in February 2026.

After three cycles of amrubicin, CT revealed a reduction in lymph node size, suggesting a favorable treatment response. The patient remained on treatment at the time of writing this manuscript (Fig. 5).

**Figure 5:**
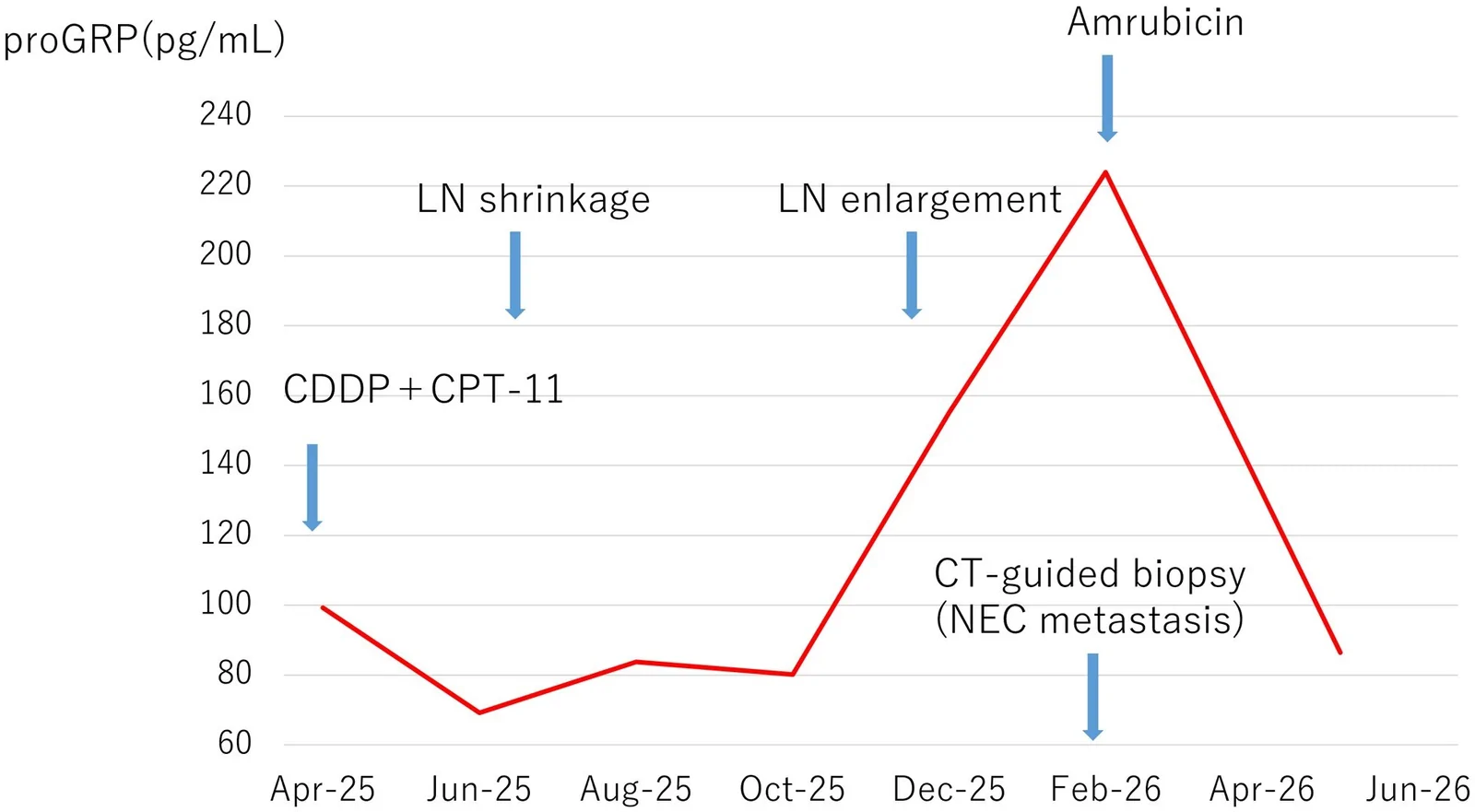
Clinical course and changes in the serum proGRP levels. CDDP, cisplatin; CPT-11, irinotecan; CT, computed tomography; LN, lymph node; NEC, neuroendocrine carcinoma.

Comprehensive genomic profiling of the adrenal tumor demonstrated a high tumor mutational burden (TMB-high). If further disease progression occurs, immune checkpoint inhibitor therapy is considered a potential therapeutic option.

## Discussion

Primary adrenal NEC is an exceptionally rare malignancy, and the small cell subtype is particularly uncommon [1]. Owing to its rarity, the clinical characteristics, optimal management strategies, and prognostic factors of primary adrenal NEC remain poorly defined [1, 2]. The present case is notable not only because of the rarity of primary adrenal small cell NEC but also because postoperative assessment of lymph node disease was complicated by the coexisting sarcoidosis and synchronous ascending colon adenocarcinoma.

The adrenal lesion in this patient was detected initially as an incidentaloma and monitored radiologically every 12 months for several years before surgical resection. Although adrenal incidentalomas are increasingly encountered in clinical practice, differentiating benign from malignant lesions remains challenging [3, 4]. Tumor enlargement during follow-up is considered an important indicator of malignancy and frequently prompts surgical intervention [3]. In the present case, the lesion demonstrated progressive growth from 7 to 24 mm × 23 mm over several years despite the absence of hormonal hypersecretion, which led to adrenalectomy. Subsequently, histopathological examination established the diagnosis of small cell NEC with a Ki-67 labeling index of 80%, indicating a highly proliferative and biologically aggressive neoplasm [2].

An important diagnostic consideration in patients with adrenal NEC is the exclusion of metastatic disease from an extra-adrenal primary site. Small cell NECs arising in the adrenal gland share histological features with small cell lung carcinoma and other extrapulmonary NECs, making determination of the primary site based on pathology alone challenging. Therefore, comprehensive clinical and radiological evaluations are essential. In the present case, chest CT revealed no pulmonary lesions suggestive of primary lung cancer, supporting the diagnosis of primary adrenal small cell NEC.

A particularly challenging aspect of this case was the interpretation of the postoperative para-aortic lymphadenopathy. FDG-PET/CT revealed marked FDG uptake within the para-aortic lymph nodes; however, the patient had a history of sarcoidosis. Sarcoidosis is a granulomatous disease that frequently demonstrates intense FDG uptake and may closely mimic metastatic malignancy on PET/CT [5-7]. Although FDG-PET/CT is widely used for staging and response assessment in malignant tumors, its specificity may be limited in patients with inflammatory or granulomatous disorders [5-7]. Consequently, distinguishing metastatic disease from active sarcoidosis based on imaging findings only might be difficult.

The coexistence of ascending colon adenocarcinoma further complicated the diagnosis. FDG uptake was observed in the ascending colon, and colonoscopic biopsy confirmed the presence of adenocarcinoma. Therefore, the possibility that the para-aortic lymphadenopathy represented metastatic colon cancer had to be considered. Nevertheless, the anatomical distribution of the enlarged lymph nodes was atypical of the usual metastatic pattern of ascending colon cancer. In addition, the serum ACE and sIL-2R levels remained stable, providing evidence against the active progression of sarcoidosis. Considering the highly aggressive pathological features of adrenal NEC and early appearance of lymphadenopathy after surgery, metastatic adrenal NEC was deemed the most likely diagnosis.

As pathological confirmation was not obtained at the time of the initial recurrence, treatment decisions were initially made based on clinical and radiological findings. The patient received cisplatin and irinotecan, a regimen commonly used for small cell lung cancer and extrapulmonary high-grade NEC [2]. An initial radiological response and a reduction in serum proGRP levels were consistent with a treatment response. However, the disease progressed after 6 cycles of treatment. CT-guided biopsy of the para-aortic lymph node was performed to differentiate recurrent NEC from progressive sarcoidosis. Histopathological examination confirmed the presence of metastatic NEC. This highlights the importance of tissue diagnosis when imaging findings are potentially confounded by coexisting inflammatory diseases. The present case illustrates the limitations of relying solely on imaging studies for disease assessment in patients with sarcoidosis and emphasizes the value of pathological confirmation whenever feasible.

Platinum-based chemotherapy remains the standard first-line treatment for high-grade NEC and is generally modeled after the therapeutic strategies used for small cell lung cancer [2]. Although the patient initially responded to cisplatin plus irinotecan, early disease progression reflected the aggressive biological behavior typically observed in small cell NEC. Second-line treatment with amrubicin resulted in tumor shrinkage and disease control at the latest follow-up.

Another noteworthy feature of this case was the identification of a TMB-high using comprehensive genomic profiling. In recent years, immune checkpoint inhibitors have emerged as a therapeutic option for select patients with TMB-high solid tumors [8]. Although the efficacy of immunotherapy in high-grade NEC remains incompletely established, durable responses have been reported in a subset of patients with TMB-high disease [8, 9]. Regarding rare tumors, such as primary adrenal NEC, comprehensive genomic profiling may provide clinically meaningful information and expand future treatment options when standard therapies are limited [9]. In the present case, immune checkpoint inhibition may represent a potential therapeutic strategy if further disease progression occurs.

This case highlights several important clinical challenges associated with primary adrenal small cell NEC. First, primary adrenal NEC should be considered in the differential diagnosis of enlarging nonfunctioning adrenal incidentalomas. Second, the interpretation of FDG-avid para-aortic lymphadenopathy might be particularly challenging in patients with coexisting sarcoidosis and synchronous ascending colon adenocarcinoma. Finally, this case underscores the importance of pathological confirmation when the etiology of recurrent lymphadenopathy is uncertain, as an accurate diagnosis directly influences treatment decisions. The accumulation of additional cases, together with comprehensive genomic profiling, may improve our understanding of the clinical characteristics of this rare malignancy and facilitate the development of more effective treatment strategies.

## Learning points

- Neuroendocrine carcinoma should be considered in enlarging nonfunctioning adrenal incidentalomas.
- Sarcoidosis may mimic metastatic lymphadenopathy on positron emission tomography/computed tomography.
- Histopathological confirmation is essential for establishing the definitive diagnosis of adrenal malignancy.
- Comprehensive genomic profiling may expand the therapeutic options.

## Contributors

All authors made individual contributions to authorship. A.S.: management of the patient, collection of clinical data, review of literature, preparation of figures, and drafting of the manuscript. T.T., A.M., and Y.N.: diagnosis and clinical management of the patient. T.Y.: responsible for the patient's surgeries. Y.K.: histopathological section and preparation of histology images. All authors reviewed and approved the final draft.

## Data Availability

Data sharing is not applicable to this article as no datasets were generated or analyzed during the current study.
